# Benefits of a virtual environment program at the level of functional physical fitness in non-institutionalized elderly

**DOI:** 10.1080/07853890.2021.1896441

**Published:** 2021-09-28

**Authors:** Ana Freitas, Ana Pacifico, Catarina Costa, Margarida Almeida, Ângela Maria Pereira

**Affiliations:** a Department of Physiotherapy, Escola Superior de Saúde Egas Moniz (ESSEM), Egas Moniz Cooperativa de Ensino Superior, Caparica, Portugal; b Centro de Investigação Interdisciplinar Egas Moniz (CiiEM), Egas Moniz Cooperativa de Ensino Superior, Caparica, Portugal; c Hospital Garcia de Orta, Almada, Portugal

## Abstract

**Introduction:**

Balance is one of the main concerns in the elderly population since there is a decline in the somatosensory system functions which may lead to a high probability of falling [1]. The risk of falling is one of the main problems among the elderly population due to its multifactorial causes [2]. One strategy to promote greater adherence and motivation to intervention in Physical Therapy is the use of virtual environment programs. This associated with a balance exercise program is an effective method for preventing falls, because it improves balance levels [3]. The purpose of this study is to analyse the benefit of a virtual environment exercise program in non-institutionalized elderly at the end of six weeks.

**Materials and methods:**

In this randomised controlled trial 68 non-institutionalized elderly from a day care institution in Corroios, were included. Thirty two subjects, age 80.6 ± 7.0 yrs constituted the experimental group (EG); and 36, age, 81.7 ± 7.1 yrs constituted the control group (CG). The EG was submitted to 6 weeks of a virtual environment exercise program performed on a Nintendo Wii, and to a set of recreational activities. The CG only performed the activities. The instruments used in the present study to evaluate performance were Tinetti’s index which evaluates the static balance and the gait to quantify the risk of fall, and the Fullerton’s functional fitness tests to assess physical parameters such as strength, aerobic endurance, flexibility and agility/balance [4]. All subjects sign an informed consent. This study follows all the principles of the Declaration of Helsinki.

**Results:**

At the end of the 6 weeks of intervention in a virtual environment, significant improvements in upper limb strength, agility and static balance were observed. In the intragroup comparison, it was possible to verify improvements in all tests of the battery of physical fitness. The values of the functional fitness test were significantly different (*p* < .05) between EG and CG groups for the following variables: 30-second chair stand 14.4 ± 2.5 vs. 10.0 ± 3.4 times (*p* = .037); arm curl 16.1 ± 3.9 vs. 13.5 ± 5.9 times (*p* = .041); 8-foot up-and-go 9.2 ± 2.1 vs. 15.3 ± 6.6 sec (*p* = .021); two min. step 120.0 ± 35.8 vs. 75.3 ± 38.4 steps (*p* = .016), respectively; as well as for the Tinetti index.

**Discussion and conclusions:**

Performing an exercise program through a virtual environment with biofeedback can provide several benefits in the elderly population due to the provision of instant feedback. Studies suggest that an exercise program with virtual environment may be an effective tool to improve balance levels and specific components of physical fitness, such as aerobic capacity, speed, agility, muscle strength and flexibility [5], which was verified in this study.
